# Ultralow-noise photonic microwave synthesis using a soliton microcomb-based transfer oscillator

**DOI:** 10.1038/s41467-019-14059-4

**Published:** 2020-01-17

**Authors:** Erwan Lucas, Pierre Brochard, Romain Bouchand, Stéphane Schilt, Thomas Südmeyer, Tobias J. Kippenberg

**Affiliations:** 1 Institute of Physics, École Polytechnique Fédérale de Lausanne (EPFL), CH-1015 Lausanne, Switzerland; 2 Laboratoire Temps-Fréquence, Université de Neuchâtel, CH-2000 Neuchâtel, Switzerland

**Keywords:** Microresonators, Frequency combs, Optical metrology, Solitons

## Abstract

The synthesis of ultralow-noise microwaves is of both scientific and technological relevance for timing, metrology, communications and radio-astronomy. Today, the lowest reported phase noise signals are obtained via optical frequency-division using mode-locked laser frequency combs. Nonetheless, this technique ideally requires high repetition rates and tight comb stabilisation. Here, a microresonator-based Kerr frequency comb (soliton microcomb) with a 14 GHz repetition rate is generated with an ultra-stable pump laser and used to derive an ultralow-noise microwave reference signal, with an absolute phase noise level below  −60 dBc/Hz at 1 Hz offset frequency and  −135 dBc/Hz at 10 kHz. This is achieved using a transfer oscillator approach, where the free-running microcomb noise (which is carefully studied and minimised) is cancelled via a combination of electronic division and mixing. Although this proof-of-principle uses an auxiliary comb for detecting the microcomb’s offset frequency, we highlight the prospects of this method with future self-referenced integrated microcombs and electro-optic combs, that would allow for ultralow-noise microwave and sub-terahertz signal generators.

## Abstract (web summary)

In order to satisfy a wide range of modern microwave applications, improved methods are needed to produce low-noise microwave signals. Here the authors demonstrate ultra-low noise microwave synthesis via optical frequency division using a transfer oscillator method applied to a microresonator-based comb on the path to future self-referenced integrated sources.

## Introduction

The synthesis of microwave signals via photonic systems, such as dual frequency lasers^1^, optoelectronic oscillators^2^, Brillouin oscillators^3^ or electro-optical dividers^4^, hold promise for their ability to synthesise low-noise or widely tunable microwave signals with compact form factor. An additional approach is based on optical frequency division, which makes use of a self-referenced fs-laser comb optically locked to an ultra-stable laser (USL) with a typical linewidth at the Hz-level^5–8^. If the comb line of index *N* is tightly phase-locked to the USL (after subtraction of the carrier envelope offset (CEO) frequency *f*_CEO_ or simultaneous stabilisation of *f*_CEO_), the comb repetition rate *f*_rep_ is directly phase-stabilised to the ultra-stable frequency *ν*_USL_ by frequency division: *f*_rep_ = *ν*_USL_∕*N*. Importantly, owing to the carrier frequency division from optics to microwaves, the absolute phase noise power spectral density is reduced by a factor *N*^2^ ~ 10^8^.

This method has been mostly implemented using fibre-based fs-lasers with repetition rates of a few hundred megahertz. A fast actuator (e.g., an intra-cavity electro-optic modulator^9^) is required to achieve a tight optical lock of the comb tooth to the optical reference and perform the frequency division over a wide bandwidth. Moreover, a high harmonic of the comb repetition rate must be used to synthesise a microwave signal beyond 10 GHz. Consequently, repetition rate multipliers are typically employed to reduce the impact of shot-noise in the photo-detection of the pulse train, such as optical filtering cavities^10^ or fibre interleavers^11^, which increases the system complexity. Therefore, the use of frequency combs directly operating at  ~10 GHz repetition rates would be highly beneficial, but their optical lock and self-referencing are challenging.

Microresonator-based Kerr frequency combs (i.e., ‘microcombs’), which naturally produce multi-GHz comb spectra generated via four-wave mixing in an optical microresonator^12,13^, are natural candidate in this context. Pumping a cavity resonance with a continuous-wave laser can initiate and sustain a circulating dissipative Kerr soliton (DKS) pulse^14–17^ that is intrinsically phase-coherent with the input pump laser. The resulting comb coupled out of the micro-cavity is inherently perfectly phase-locked to the pump laser, without any actuator locking bandwidth limitation. Direct soliton generation from an ultra-stable pump laser holds potential for compact and powerful optical-to-microwave dividers. Although self-referenced optical microcombs and clocks have been demonstrated^18–20^, optical frequency division for ultralow-noise microwave generation using such devices has not been demonstrated so far, mainly due to the complex crosstalk occurring between their two degrees of freedom^19,21^ and the limited performance of the available actuators^17,22^.

Here, we demonstrate the generation of an ultralow-noise microwave signal using a microcomb-based transfer oscillator method to realise optical-to-microwave frequency division. The transfer oscillator method^23,24^ bypasses the need for tight optical phase-locking of the frequency comb to the optical reference. Instead, it relies on an adequate manipulation and combination of signals to cancel the comb phase noise and to provide a broadband electronic division of the USL frequency to the microwave domain. The frequency division by a large factor *N* is performed electronically, thus removing the need for high locking bandwidth actuators. In this work, the USL is used to pump the microresonator and inherently constitutes a tooth of the resulting frequency comb. We show how to extend the transfer oscillator technique to exploit this salient feature of microcombs (or equivalently of electro-optic combs^25^). In this proof-of-principle demonstration, we achieved a measured single-sideband phase noise of  −110 dBc/Hz at 200 Hz offset from the 14.09-GHz carrier, which is 15 dB below the lowest phase noise microresonator-based photonic oscillator reported so far^26^, demonstrating the potential of this approach.

## Results

### Transfer oscillator principle

**Fig. 1:**
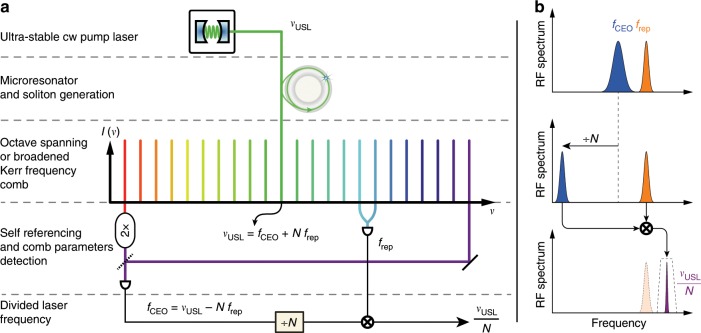
Principle of operation of the Kerr comb-based transfer oscillator. for optical-to-microwave frequency division. **a** Schematic illustration of the transfer oscillator applied to a Kerr comb (or electro-optic combs equivalently). **b** Schematic representation of the signal evolution along the electronic division chain leading to the low-noise output signal. The two comb parameters *f*_CEO_ and *f*_rep_ are detected. Both parameters can be free-running and fluctuate. The carrier envelope offset (CEO) frequency is electronically divided by a large number *N* that corresponds to the tooth number of the ultra-stable pump *ν*_USL_. After this step, the frequency fluctuations of the divided CEO *f*_CEO_∕*N* = *ν*_USL_∕*N* − *f*_rep_ are dominated by the repetition rate fluctuations. These are removed by mixing *f*_CEO_∕*N* with *f*_rep_ to obtain the division result *ν*_USL_∕*N*. A narrow-band filtering is used to reject spurs.

The high-level working principle of our method is illustrated in Fig. 1. A microresonator pumped by a sub-Hz-linewidth USL at frequency *ν*_USL_ generates a soliton-Kerr comb with a GHz-range repetition rate *f*_rep_ that is set by the resonator free spectral range (FSR). The reference laser is part of the frequency comb (line *N*) such that its frequency can be written as *ν*_USL_ = *f*_CEO_ + *N* *f*_rep_. The detection of the CEO frequency (for example via *f* − 2*f* interferometry^27,28^ or with an auxiliary self-referenced comb as in the present work) is followed by electronic division by means of a combination of frequency pre-scalers and direct digital synthesisers (DDS). The final step consists of mixing the divided CEO signal with the repetition rate, which yields

1 $${f}_{{\rm{signal}}}=\,\frac{{f}_{{\rm{CEO}}}}{N}+{f}_{{\rm{rep}}}=\frac{{\nu }_{{\rm{USL}}}}{N}$$

Importantly, this process can be carried out with a free-running Kerr comb and circumvents the need for a high-bandwidth repetition rate lock.

### Microcomb generation with the USL

**Fig. 2:**
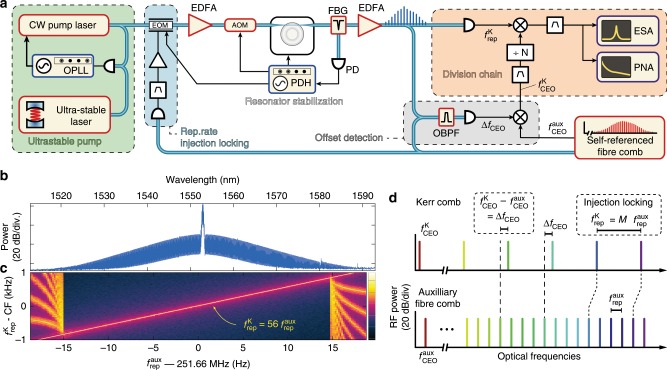
Experimental setup and CEO detection with the auxiliary comb. **a** Setup for Kerr comb-based optical frequency division. The details of each highlighted block can be found in Supplementary Notes 1–5. EDFA, Er-doped fibre amplifier; AOM, Acousto-optic modulator; EOM, Electro-optic modulator; FBG, Fibre Bragg grating for pump rejection; OBPF, Optical band-pass filter; PD, Photodiode; OPLL, Optical phase-lock loop; PDH, Pound-Drever-Hall lock; PNA, Phase noise analyser; ESA, Electrical spectrum analyser. **b** Optical spectrum of the soliton-based Kerr comb, prior to pump suppression. (**c**) Radio-frequency (RF) spectrogram showing the injection-locking effect of the Kerr comb repetition rate ${f}_{{\rm{rep}}}^{{\rm{K}}}$ to the 56th harmonic of the auxiliary comb repetition rate ${f}_{{\rm{rep}}}^{{\rm{aux}}}$, obtained by changing the frequency of ${f}_{{\rm{rep}}}^{{\rm{aux}}}$. Here, the harmonic power applied to the EOM is  ~11 dBm yielding a locking range of  ~1.7 kHz. The spectrum of ${f}_{{\rm{rep}}}^{{\rm{K}}}$ is centred at CF = 14.092943 GHz and the resolution bandwidth is 5 Hz. **d** Principle of the Kerr comb CEO detection with the auxiliary comb. The harmonic relation between the repetition rate of both combs is ensured via injection locking for *M* = 56. The heterodyne beat between the two combs thus yields the difference between their carrier-envelope offset frequency (*Δf*_CEO_).

Due to the limited tuning of the resonator and narrow bandwidth of the microcomb used for this proof-of-concept, additional hardware was needed to demonstrate the transfer oscillator principle, as shown in Fig. 2a. The soliton-Kerr comb is generated by pumping a crystalline magnesium fluoride (MgF_2_) microresonator with an FSR of 14.09 GHz using a 1553-nm diode laser, which is initially quickly scanned across a resonance for soliton generation^14^. Next, the pump laser is phase-locked to a sub-Hz-linewidth USL (Menlo Systems ORS1500) at a frequency detuning of  ~1.7 GHz, thereby acquiring a comparable level of purity and stability (green box in Fig. 2a, a detailed description is provided in the Supplementary Note 1).

To ensure the long-term stable operation of the Kerr comb and prevent the decay of the soliton, the microresonator resonance is slowly locked to the pump laser with an effective-detuning stabilisation, achieved via a sideband Pound-Drever-Hall (PDH) lock^29^, which feedbacks on an acousto-optic modulator (AOM) that modulates the pump power and thus thermo-optically tunes the resonator, in addition to a slow thermal actuation of the microresonator (see details in the Supplementary Note 2). The detuning setpoint was carefully optimised in order to minimise the noise of the Kerr comb repetition rate ${f}_{{\rm{rep}}}^{{\rm{K}}}$ at offset frequencies beyond  ~100 Hz (see the Methods section on noise limitations). However, the residual thermal drift of the resonator degrades the performance at lower offset frequencies.

### Offset detection with an auxiliary comb

The used crystalline MgF_2_ micro-comb features a relatively narrow spectrum that prevents a direct detection of its CEO frequency (Fig. 2b). The self-referencing of Kerr combs remains highly demanding due to the high repetition rate, low optical power, and fairly long pulse duration (225 fs here) resulting in a low peak intensity that makes the spectral broadening for *f* − 2*f* interferometry challenging^19,30^. Therefore, we implemented an indirect detection scheme using an auxiliary self-referenced fibre-laser frequency comb^31^ with a repetition rate ${f}_{{\rm{rep}}}^{{\rm{aux}}}=251.7$ MHz, as schematised in Fig. 2d. Provided the repetition rates of both combs are harmonically phase-locked, i.e., ${f}_{{\rm{rep}}}^{{\rm{K}}}=M\ {f}_{{\rm{rep}}}^{{\rm{aux}}}$ (superscripts ‘K’ and ‘aux’ refer to the Kerr and auxiliary combs, respectively), then the optical beatnote between the two combs corresponds to their relative CEO frequency $\Delta {f}_{{\rm{CEO}}}={f}_{{\rm{CEO}}}^{{\rm{K}}}-{f}_{{\rm{CEO}}}^{{\rm{aux}}}$, as the repetition rate noise contributions compensate each other in this beat signal. The Kerr comb CEO frequency is then obtained by mixing out the CEO frequency of the auxiliary comb ${f}_{{\rm{CEO}}}^{{\rm{aux}}}$ detected with an *f* − 2*f* interferometer (see Fig. 2a) and corresponds to ${f}_{{\rm{CEO}}}^{{\rm{K}}}=\Delta {f}_{{\rm{CEO}}}+{f}_{{\rm{CEO}}}^{{\rm{aux}}}={\nu }_{\text{pump}}-N{f}_{{\rm{rep}}}^{{\rm{K}}}$ (grey box in Fig. 2a). Importantly, the auxiliary comb is not stabilised to the USL at any point and thus does not perform the division. Its role is limited to the offset detection in this demonstration.

The mutual harmonic phase-locking of the comb repetition rates is achieved via soliton injection-locking^32^. The harmonic *M* = 56 of the repetition rate of the auxiliary comb (at 14.093 GHz) is detected, filtered and amplified to phase-modulate the pump light using an electro-optic modulator (EOM, blue box in Fig. 2a). This frequency is very close to the native microcomb line spacing, which gets injection-locked to this drive signal. Therefore, both repetition rates are strongly correlated over a bandwidth of  ~2 kHz (see the Supplementary Note 4 and Supplementary Fig. 3).

### Transfer oscillator chain

**Fig. 3:**
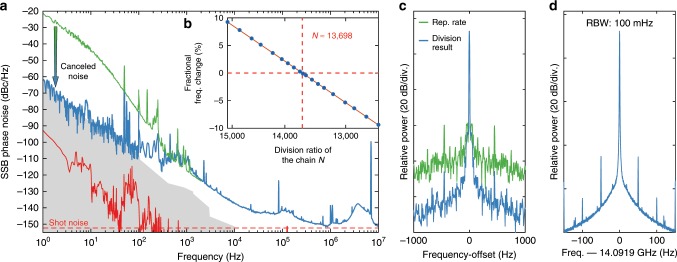
Characterisation of the optical-to-microwave division signal. **a** Absolute single-sideband (SSB) phase noise of the 14.09 GHz signal generated by optical-to-microwave division of the USL via the Kerr comb transfer oscillator (blue) and obtained directly from the Kerr comb repetition rate (green) for comparison. The sensitivity limit of the phase noise analyser (3000 cross correlations applied at 1 Hz) is indicated by the grey shaded area. The red line is the limit inferred from the optical phase noise of the USL^71^, assuming an ideal noiseless division. **b** Precise determination of the optimal division factor *N* corresponding to the zero crossing of the linear fit (solid line) of the measured relative frequency change of the generated RF signal for a small variation of the repetition rate (dots). **c** Comparison between the RF spectra of the Kerr comb repetition rate and the optical-to-microwave frequency division result. The resolution bandwidth (RBW) is 5 Hz. **d** RF spectrum of the frequency-divided output signal, the RBW is 100 mHz. The data was acquired with the IQ demodulation mode of the spectrum analyser.

The Kerr comb CEO signal, indirectly obtained as previously described, is detected at low frequency (MHz-range) and filtered to match the bandwidth of the injection locking of the repetition rate (not represented in Fig. 2a, see the Supplementary Note 5 and Supplementary Fig. 4). After up-mixing to 15 GHz, it is frequency-divided by a large pre-determined factor *N* ≈ 13,698 and is subtracted to the separately-detected repetition rate ${f}_{{\rm{rep}}}^{{\rm{K}}}$ to obtain the frequency-divided signal of the ultra-stable pump laser: ${\nu }_{\text{pump}}/N={f}_{{\rm{CEO}}}^{{\rm{K}}}/N+{f}_{{\rm{rep}}}^{{\rm{K}}}$ (orange box in Fig. 2a). The overall division of the Kerr comb CEO signal by the factor *N* is realised with a frequency pre-scaler followed by two parallel DDS, which offers improved filtering capabilities in the electronic division^24^. This second stage division with the DDS allows for a precise non-integer frequency division factor and leads to a clean single-tone output signal corresponding to the frequency-divided USL (see Fig. 3d). The detailed description of the frequency division chain is provided in the Supplementary Note 5.

The overall division factor *N* was accurately determined experimentally, without prior knowledge of the optical frequency of the ultra-stable pump laser, by measuring the frequency change of the generated microwave signal corresponding to a small variation (140 Hz) of the Kerr comb repetition rate for different programmed division factors *N* (see Fig. 3b). This simple measurement also provides an accurate determination of an optical comb line index *N* that can be useful for absolute optical frequency measurements.

### Microwave characterisation

The phase noise of the generated ultralow-noise 14.09 GHz signal was measured with a cross-correlator phase noise analyser (Fig. 3a). It reaches  −110 dBc/Hz at 200 Hz Fourier frequency, 15 dB below the lowest phase noise microresonator-based photonics oscillator^26^ at 10 GHz. The phase noise is below  −135 dBc/Hz at 10 kHz and  −150 dBc/Hz at around 1 MHz, showing that the intrinsic low-noise properties of the soliton Kerr comb at high Fourier frequencies are preserved. The calculated shot-noise predicts a noise floor at  −152 dBc/Hz (thermal noise floor  ~ −170 dBc/Hz). At 1 Hz offset, the measurement is limited by the instrumental noise floor below  −60 dBc/Hz, even with 3000 cross correlations. Nevertheless, the transfer oscillator offers an improvement by at least 40 dB compared to the direct detection of the Kerr comb repetition rate (despite the resonator being stabilised to the USL), showing its ability to cancel the residual thermal drifts of the Kerr cavity. The technical limitations of the measurement, at low offset frequency, make it difficult to directly compare the method with state-of-the-art optical frequency-division using mode-locked lasers. Nevertheless, at high Fourier frequencies, our results surpass some of the first demonstrations of optical frequency division^5^, even if no optimisation has been performed on the photodetection side. Over the past 10 years, the development of mode-locked lasers, as well as the improvement of photodetection noise^33,34^, led to a reduction of the noise of the generated microwaves by 30–40 dB in some frequency bands^8^. We believe that the transfer oscillator method can follow a similar path, as in particular, the high repetition rates of the Kerr combs should make the photodetection optimisation less stringent.

## Discussion

In summary, we have reported optical-to-microwave frequency division using a Kerr comb as transfer oscillator. This demonstrates the potential of this method in microwave photonics and enlarges its previously reported implementation with low repetition rate mode-locked lasers. The approach presented here can be further implemented with electro-optic combs, where self-referencing and feedback control were recently achieved^25,35^. Although this proof-of-principle experiment required an auxiliary comb to obtain the CEO frequency of the Kerr comb, directly self-referenced microcombs are technologically feasible in silicon nitride (Si_3_N_4_) photonic-chips^36^. While octave-spanning comb spectra have been achieved using dispersion control^20,37^, these implementations used THz repetition rates to cover such a large spectral range, which made photodetection of the repetition rate practically impossible. Nonetheless, the residual phase noise of these combs has been shown to be suitable for frequency division^38^. Recent improvements of integrated resonators have enabled soliton microcombs with K- and X-band (20 and 10 GHz) repetition rates in integrated resonators^39^. However, the achieved spectral spans, although wider than in the crystalline case, are far from covering one octave. Pulsed pumping^40^ appears as a promising approach to enable octave-spanning microcombs with detectable microwave repetition rates. This approach uses synchronous pumping of the microresonator with picosecond pulses to generate a soliton with a much shorter duration and a spectrum that can cover an octave, similar to enhancement cavities^41^. It can be seen as a hybrid between an electro-optic (EO) comb and a microcomb, with the advantage that the spectral enlargement of the EO comb is performed in cavity and is therefore directly filtered^42,43^. Crucially, even if the free-running phase noise of these integrated microcombs is typically higher than in the crystalline platform used in this work^39,44,45^, the additional noise is cancelled over a broad frequency range via the transfer oscillator method that constitutes a powerful tool for low-noise frequency division without the need for a very low-noise comb. The free-running comb operation and the maturity of RF components, which can be suitably integrated, promise robust device operation. Furthermore, improvements in resonator actuation, using micro-heaters^17^, piezoelectric transducers^46–48^ or the electro-optic effect^49,50^, will allow the resonator to be tuned to the USL for direct soliton generation (as in Fig. 1a), alleviating the need for an optical phase-lock loop and greatly simplifying the detuning stabilisation mechanism. If a lower stability level is acceptable, simpler and more compact low-noise lasers can be employed^51–53^ instead of the USL. We believe that the presented transfer oscillator method holds promising potential for ultralow-noise high-frequency generators with a new generation of compact photonic-based systems^54^ for radar applications^55^, high-frequency telecommunications^56^ and time-frequency metrology^5^.

## Methods

### Operating conditions

The pump power after the EOM used for the PDH lock of the microresonator and the injection locking of *f*_rep_ is  ~10 mW and is amplified to  ~250 mW in an EDFA. The power level after the AOM that controls the pump power coupled to the resonator (see Fig. 2a) is set to  ~210 mW. After comb generation and residual pump rejection with a fibre Bragg grating, the comb power of  ~1 mW is amplified to  ≳5 mW. The largest part of this power (90%) is sent onto a high power handling photodiode (Discovery Semiconductors DSC40S, generating a photocurrent of 5.12 mA and a microwave power of  ~−7.4 dBm), while the remaining fraction is used for the intercomb beatnote detection. The shot-noise level is estimated for a CW laser detection, based on the generated photocurrent and microwave power. The 56th harmonic of the auxiliary comb repetition rate ${f}_{{\rm{rep}}}^{{\rm{aux}}}$ at 14.09 GHz is detected, selected using a narrow band-pass filter and amplified to  ~19 dBm. This signal drives the phase modulator and creates an estimated phase deviation of  ~1.4  rad. The injection-locking range of the Kerr comb repetition rate^32^ spans  ≳2 kHz and the locking bandwidth is  ~2 kHz.

### Resonator characteristics

The MgF_2_ whispering gallery mode resonator was fabricated via precision diamond turning and hand polishing on a lathe. The intrinsic linewidth of the pumped mode is  ~80 kHz (intrinsic quality factor of 2.4 × 10^9^). The evanescent coupling to the resonance is achieved via a tapered optical fibre. The fibre is operated in contact with the resonator to damp its vibrations. Careful adjustment of the fibre position is required to maximise the coupling rate and increase the out-coupled comb power. The loaded resonance linewidth is estimated at  ~2.4 MHz. The threshold power for comb formation is estimated at  ~40 mW. The detuning setpoint was chosen to minimise the noise of the Kerr comb repetition rate, as described in the next section.

### Soliton noise minimisation

The laser-resonator detuning *δ* = *ν*_cav_ − *ν*_laser_ is known to have a major impact on the noise and stability of Kerr frequency combs. This parameter not only sets the soliton pulse duration^57^, but was also shown to modify the repetition rate frequency through the Raman self-frequency shift^58^ Ω_Raman_(*δ*) and the soliton recoil Ω_recoil_ corresponding to dispersive wave emission^57,59^. Indeed, these two effects lead to an overall shift of the spectral centre of the soliton (i.e., the soliton spectral maximum relative to the pump frequency) Ω = Ω_Raman_ + Ω_recoil_, which induces in turn a change in the group velocity of the pulse and therefore of the repetition rate according to ref. ^60^

2 $${f}_{{\rm{rep}}}^{{\rm{K}}}=\frac{1}{2\pi }\left({D}_{1}+\frac{{D}_{2}}{{D}_{1}}\ \Omega (\delta )\right)$$

where *D*_1_∕2*π* = 14.09 GHz is the resonator FSR and *D*_2_∕2*π* = 1.96 kHz is the group velocity dispersion (GVD) parameter at the pump frequency^61^. Thus, residual laser-resonator detuning noise can degrade the spectral purity of the repetition rate^21^. A solution to this problem was already identified by Yi et al.^59^, who proposed to use the balance of dispersive-wave recoil and Raman-induced soliton-self-frequency shift to enhance the repetition-rate stability of a silica wedge-based Kerr comb. A similar concept is applied here to minimise the repetition rate noise of the crystalline MgF_2_ microresonator-based comb. Importantly, in MgF_2_, the Raman self-frequency shift can be neglected, due to the very narrow gain bandwidth, and the soliton shift is dominated by the soliton recoil Ω  ≈ Ω_recoil_.

**Fig. 4:**
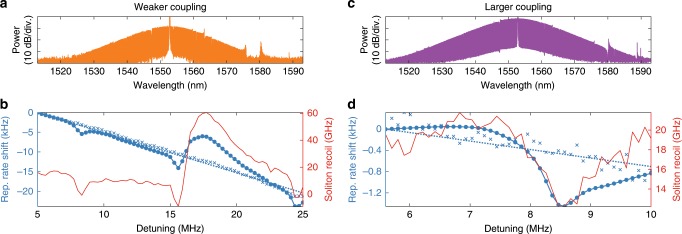
Optimisation of ${f}_{{\rm{rep}}}^{{\rm{K}}}$ phase noise. **a** Soliton spectrum for lower coupling case (Detuning 10 MHz). **b** Evolution of the repetition rate (blue, solid) and of the soliton recoil (Ω∕*2π*) retrieved by fitting the optical spectrum (red), in the lower coupling case. The blue crosses and dashed line show the residual repetition rate change after subtraction of the recoil induced shift (using Eq. (2)). **c** Soliton spectrum for larger coupling case (Detuning 10 MHz). **d** Evolution of the repetition rate (blue, solid) and of the soliton recoil (Ω∕2*π*) retrieved by fitting the optical spectrum (red), in the larger coupling case. The blue dashed line shows the residual repetition rate change after subtraction of the recoil induced shift (using Eq. (2)).

We measured the variation in repetition rate of the soliton comb as a function of detuning in two coupling conditions (weak and large coupling). The coupling rate was modified by changing the position of the tapered fibre along the resonator. The detuning was scanned (forward and backward) by changing the PDH modulation frequency, while the phase-lock loop offset frequency was adapted accordingly to keep the total frequency offset between the USL and the microresonator resonance constant. At each detuning point, the optical spectrum was acquired and the repetition rate frequency ${f}_{{\rm{rep}}}^{{\rm{K}}}$ was counted. The results are displayed in Fig. 4. The phase modulation at the cavity FSR used for injection-locking was disabled in this measurement.

The weak coupling of the resonator allows for a relatively wide detuning range to be accessed (5–25 MHz, see Fig. 4b). Over this span, the repetition rate changes in total by 22 kHz, but not linearly. The non-monotonic evolution of ${f}_{{\rm{rep}}}^{{\rm{K}}}(\delta )$ is caused by the soliton recoil induced by dispersive waves through avoided mode crossings^57,59,62^. The soliton shift *Ω*∕2*π* is extracted by fitting the optical spectrum with a ${{\rm{sech}} }^{2}$ function and the associated repetition rate variation can be estimated using Eq. (2). Interestingly, after subtracting this contribution, the residual shift of the repetition rate follows a linear trend with a slope of  ~−1 kHz/MHz. This significant variation is independent from any recoil-associated effect and could originate from more complex forms of avoided modal crossings, or third order dispersion, although we observed that the value of this slope changes with the coupling as detailed below.

Increasing the coupling rate of the resonator (see Fig. 4d) shrinks the accessible detuning range (5.5–10 MHz) and radically changes the dependence of ${f}_{{\rm{rep}}}^{{\rm{K}}}$ with *δ*. The overall variation is reduced to  ~ 1.4 kHz and is dominated by solitonic recoil. Once this contribution is subtracted, the residual slope is on the order of  ~−60 Hz/MHz, which is very close to the value expected from the nonlinear self-steepening effect^63^.

### Quiet operation point

**Fig. 5:**
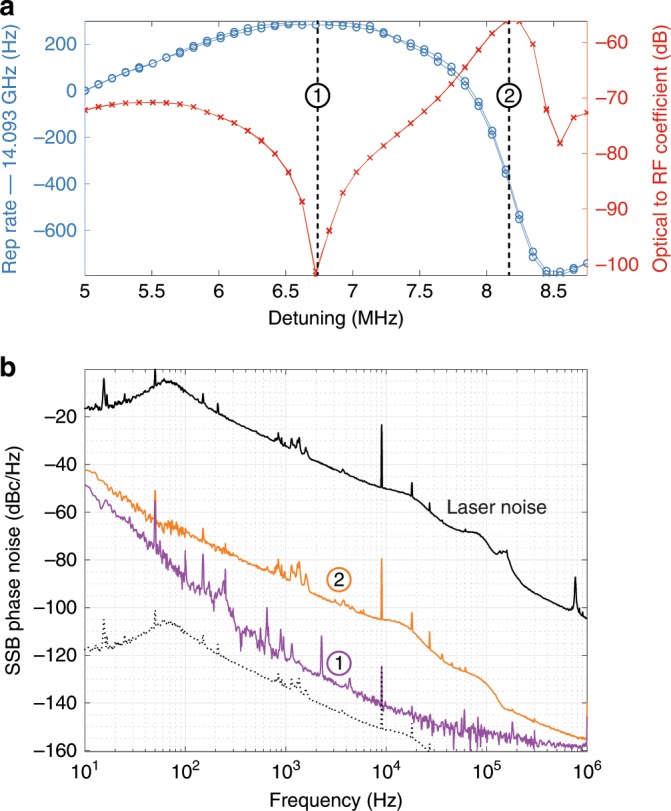
Quiet operating point. **a** Evolution of the repetition rate with the detuning (blue) and associated optical phase modulation to RF phase modulation conversion coefficient calibrated with the 9-kHz phase modulation tone on the laser (red). **b** Phase noise spectra of the soliton repetition rate at the two operating points highlighted in (**a**). The solid black line shows the laser noise (PDH-stabilised to the microcavity). The dashed black line shows the noise of the laser scaled by  −100 dB to match the 9-kHz phase calibration tone.

More notably, under this larger coupling condition, the relation ${f}_{{\rm{rep}}}^{{\rm{K}}}(\delta )$ exhibits a stationary point around *δ* = 7 MHz, where the coupling of pump-laser frequency noise into the soliton repetition rate is expected to be minimal since $\partial {f}_{{\rm{rep}}}^{{\rm{K}}}/\partial \delta \approx 0$. To verify this prediction, the phase noise of the detected soliton pulse train was measured at different detuning points. The pump laser was phase-modulated by a low frequency tone at 9 kHz to provide a reference point. Furthermore, instead of phase-locking the pump laser to the USL, the PDH feedback was applied to the pump laser current in these measurements, and the resonator was slowly stabilised to the USL via power and thermal feedback. The larger laser noise obtained in this case helps visualising its impact on the repetition rate frequency and could be calibrated via a heterodyne measurement with the USL. The results are displayed in Fig. 5. At the operating point 2, where the slope of ${f}_{{\rm{rep}}}^{{\rm{K}}}(\delta )$ is maximum, the noise of ${f}_{{\rm{rep}}}^{{\rm{K}}}$ follows the same features as the laser noise. Rescaling the laser noise to match the 9 kHz modulation peaks indicates that the optical noise is reduced by 56 dB. Conversely the point 1, where the slope of ${f}_{{\rm{rep}}}^{{\rm{K}}}(\delta )$ is minimum, corresponds to the lowest optical-to-RF noise transduction (dip in Fig. 5a), with a conversion coefficient below  −100 dB. As expected, this point yields the lowest achieved phase noise, and it appears that the laser phase noise is no longer the overall limiting factor of the Kerr comb repetition rate noise.

### Noise limitations in microcombs

**Fig. 6:**
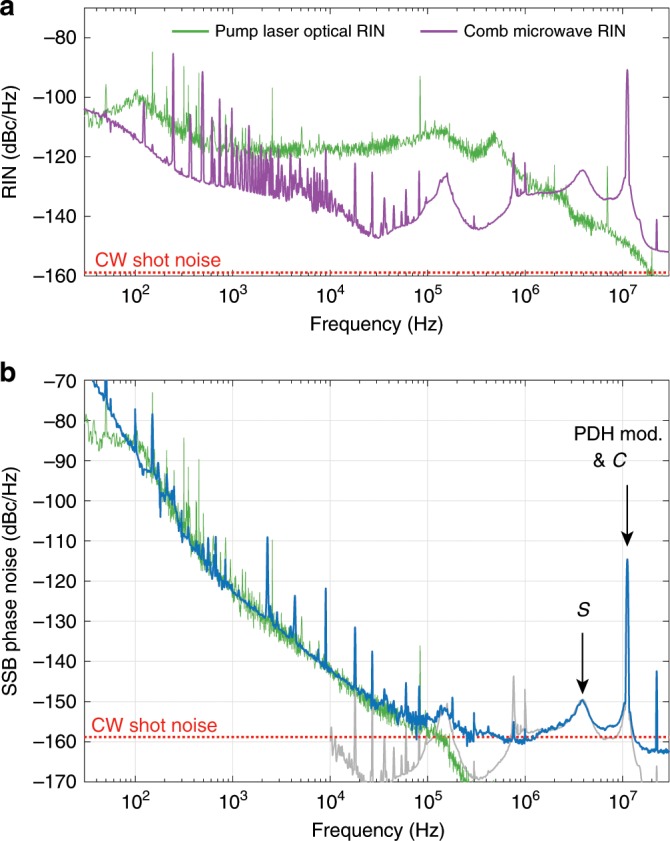
Pump laser RIN and estimated limitation on the phase noise. **a** Optical RIN of the pump laser (green) and microwave amplitude noise of the soliton repetition rate (purple). **b** Phase noise spectrum of the repetition rate in the quiet point (blue) and estimated limitation from the pump laser RIN (green). The grey curve corresponds to the estimated AM-to-PM conversion in the photodiode (microwave amplitude noise scaled by  −25 dB).

In a nonlinear resonator, the free spectral range *D*_1_∕2*π* depends on the circulating optical power. Therefore, the relative intensity noise (RIN) of the pump laser (power *P*_in_) eventually induces timing jitter of the repetition rate, according to Eq. (2). Assuming a laser on resonance, the self phase modulation induced shift follows^64^:

3 $$\frac{\delta {D}_{1}(\omega )}{2\pi }=\underbrace{\left(\frac{{D}_{1}}{2\pi }\frac{4\eta c{n}_{2}}{\kappa {V}_{{\rm{eff}}}{n}_{0}^{2}}\right)}_{{\alpha}}\ \delta {P}_{{\rm{in}}}(\omega )$$

where *κ*∕2*π* ≈ 1.35 MHz is the cavity energy decay rate, *η* = *κ*_ex_∕*κ* ≈  0.94 is the coupling impedance of the resonator (*κ*_ex_ is the coupling rate), *V*_eff_ ≈ 2.32 × 10^−12^ m^3^ is the mode volume, *n*_2_ = 9 × 10^−21^ m^2^∕W is the (Kerr) nonlinear index and *n*_0_ = 1.37 is the refractive index. These values yield a conversion coefficient *α* ≈ 3.8 kHz∕W. We measured the relative intensity noise *S*_RIN_(*f*) of the pump laser (Fig. 6a) and the associated induced phase noise was estimated using:

4 $${S}_{{D}_{1}/2\pi }^{\phi }(f)={\left(\frac{\alpha }{f}{P}_{{\rm{in}}}\right)}^{2}{S}_{{\rm{RIN}}}(f)$$

for the measured input pump power of *P*_in_ ≈ 212 mW. The results are displayed in Fig. 6b. The estimated level matches remarkably the repetition rate phase noise at offsets between 500 Hz and 100 kHz (blue and green curves in Fig. 6b), suggesting that the pump laser RIN is limiting the performances in this range. The phase noise reaches  ~−143 dBc/Hz at 10 kHz, which outperforms any other microresonator-based approach^3,4,26,46,59^.

At lower offset frequencies (50–500 Hz), the thermal fluctuations and drift of the resonator, which are beyond the power stabilisation bandwidth, are the limiting factor^26,65^.

At higher offset frequencies, two noise bumps appear related to the characteristic double resonant response (${\mathcal{S}}$ and ${\mathcal{C}}$) of the resonator in the soliton regime^66^. In these resonant features, the transduction of the pump laser noise is enhanced^21^. Beyond 100 kHz offset, the contributions of various factors are more difficult to identify. We observed nonetheless a correlation between the microwave RIN (Fig. 6a) and the phase noise, which suggests that amplitude-to-phase noise conversion is occurring in the photodiode^67^, with a conversion of  ~−25 dB (grey curve in Fig. 6b), which is in agreement with reported values for similar photodiodes^34^. We report here the microwave amplitude noise, as our measurement device offered a better sensitivity in this configuration, but our observations showed that this amplitude noise matches well the optical RIN (measured at DC with a diplexer).

Finally, the continuous-wave shot-noise floor is expected to be at  −159 dBc/Hz (photocurrent of 6.85 mA, microwave power of  −3.8  dBm). However, we noticed that the phase noise floor of our measurement stands 4.1 dB below this value (at frequency offsets above 20 MHz), while the amplitude noise floor is 6.3 dB above. This imbalance between amplitude and phase needs further investigation and could be related to shot-noise correlations in the detection of optical pulses^68–70^.

## Supplementary information

Supplementary Information

## Data Availability

The data and code used to produce the results of this manuscript are available on Zenodo:10.5281/zenodo.3515211.

## References

[1] Pillet G (2008). Dual-frequency laser at 1.5 *μ*m for optical distribution and generation of high-purity microwave signals. J. Lightwave Technol..

[2] Maleki L (2011). The optoelectronic oscillator. Nat. Photonics.

[3] Li J, Lee H, Vahala KJ (2013). Microwave synthesizer using an on-chip brillouin oscillator. Nat. Commun..

[4] Li J, Yi X, Lee H, Diddams SA, Vahala KJ (2014). Electro-optical frequency division and stable microwave synthesis. Science.

[5] Millo J (2009). Ultralow noise microwave generation with fiber-based optical frequency comb and application to atomic fountain clock. Appl. Phys. Lett..

[6] Fortier TM (2011). Generation of ultrastable microwaves via optical frequency division. Nat. Photonics.

[7] Portuondo-campa E, Buchs G, Kundermann S, Balet L, Lecomte S (2015). Ultra-low phase-noise microwave generation using a diode-pumped solid-state laser based frequency comb and a polarization-maintaining pulse interleaver. Opt. Express.

[8] Xie X (2017). Photonic microwave signals with zeptosecond-level absolute timing noise. Nat. Photonics.

[9] Hudson DD (2005). Mode-locked fiber laser frequency-controlled with an intracavity electro-optic modulator. Opt. Lett..

[10] Diddams SA (2009). Improved signal-to-noise ratio of 10 ghz microwave signals generated with a mode-filtered femtosecond laser frequency comb. Opt. Express.

[11] Haboucha A (2011). Optical-fiber pulse rate multiplier for ultralow phase-noise signal generation. Opt. Lett..

[12] Gaeta AL, Lipson M, Kippenberg TJ (2019). Photonic-chip-based frequency combs. Nat. Photonics.

[13] Kippenberg TJ, Gaeta AL, Lipson M, Gorodetsky ML (2018). Dissipative kerr solitons in optical microresonators. Science.

[14] Herr T (2013). Temporal solitons in optical microresonators. Nat. Photonics.

[15] Yi X, Yang Q-F, Yang KY, Suh M-G, Vahala K (2015). Soliton frequency comb at microwave rates in a high-q silica microresonator. Optica.

[16] Brasch V (2015). Photonic chip-based optical frequency comb using soliton cherenkov radiation. Science.

[17] Joshi C (2016). Thermally controlled comb generation and soliton modelocking in microresonators. Opt. Lett..

[18] Jost JD (2015). Counting the cycles of light using a self-referenced optical microresonator. Optica.

[19] Del’Haye P (2016). Phase-coherent microwave-to-optical link with a self-referenced microcomb. Nat. Photonics.

[20] Newman ZL (2019). Architecture for the photonic integration of an optical atomic clock. Optica.

[21] Stone JR (2018). Thermal and nonlinear dissipative-soliton dynamics in kerr-microresonator frequency combs. Phys. Rev. Lett..

[22] Papp SB, Del’Haye P, Diddams SA (2013). Mechanical control of a microrod-resonator optical frequency comb. Phys. Rev. X.

[23] Telle HR, Lipphardt B, Stenger J (2002). Kerr-lens, mode-locked lasers as transfer oscillators for optical frequency measurements. Appl. Phys. B.

[24] Brochard P, Schilt S, Südmeyer T (2018). Ultra-low noise microwave generation with a free-running optical frequency comb transfer oscillator. Opt. Lett..

[25] Beha K (2017). Electronic synthesis of light. Optica.

[26] Liang W (2015). High spectral purity kerr frequency comb radio frequency photonic oscillator. Nat. Commun..

[27] Telle HR (1999). Carrier-envelope offset phase control: a novel concept for absolute optical frequency measurement and ultrashort pulse generation. Appl. Phys. B.

[28] Jones DJ (2000). Carrier-envelope phase control of femtosecond mode-locked lasers and direct optical frequency synthesis. Science.

[29] Thorpe JI, Numata K, Livas J (2008). Laser frequency stabilization and control through offset sideband locking to optical cavities. Opt. Express.

[30] Lamb ES (2018). Optical-frequency measurements with a kerr microcomb and photonic-chip supercontinuum. Phys. Rev. Appl..

[31] Tian H, Raabe N, Song Y, Steinmeyer G, Hu M (2018). High-detectivity optical heterodyne method for wideband carrier-envelope phase noise analysis of laser oscillators. Opt. Lett..

[32] Weng W (2019). Spectral purification of microwave signals with disciplined dissipative kerr solitons. Phys. Rev. Lett..

[33] Ivanov, E. N., McFerran, J. J., Diddams, S. A. & Hollberg, L. Noise properties of microwave signals synthesized with femtosecond lasers. In *Proc. 2005 IEEE International Frequency Control Symposium and Exposition, 2005*, 932–936. (IEEE, 2005). 10.1109/FREQ.2005.1574059. 10.1109/tuffc.2007.307 17441583

[34] Bouchand R, Nicolodi D, Xie X, Alexandre C, Coq YL (2017). Accurate control of optoelectronic amplitude to phase noise conversion in photodetection of ultra-fast optical pulses. Opt. Express.

[35] Carlson DR (2018). Ultrafast electro-optic light with subcycle control. Science.

[36] Brasch V, Lucas E, Jost JD, Geiselmann M, Kippenberg TJ (2017). Self-referenced photonic chip soliton kerr frequency comb. Light Sci. Appl..

[37] Pfeiffer MHP (2017). Octave-spanning dissipative kerr soliton frequency combs in si_3n_4 microresonators. Optica.

[38] Drake TE (2019). Terahertz-rate kerr-microresonator optical clockwork. Phys. Rev. X.

[39] Liu, J. et al. Nanophotonic soliton-based microwave synthesizers. http://arxiv.org/abs/1901.10372 (2019).

[40] Obrzud E, Lecomte S, Herr T (2017). Temporal solitons in microresonators driven by optical pulses. Nat. Photonics.

[41] Lilienfein N (2019). Temporal solitons in free-space femtosecond enhancement cavities. Nat. Photonics.

[42] Anderson, M. H. et al. Photonic chip-based resonant supercontinuum. **16**, 1–10, http://arxiv.org/abs/1909.00022 (2019).

[43] Brasch V, Obrzud E, Lecomte S, Herr T (2019). Nonlinear filtering of an optical pulse train using dissipative kerr solitons. Optica.

[44] Huang G (2019). Thermorefractive noise in silicon-nitride microresonators. Phys. Rev. A.

[45] Drake, T. E., Stone, J. R., Briles, T. C. & Papp, S. B. Thermal decoherence and laser cooling of kerr microresonator solitons. 1–16, http://arxiv.org/abs/1903.00431 (2019).

[46] Liang W (2017). Stabilized *C*-band kerr frequency comb. IEEE Photonics J..

[47] Liu, J. et al. Monolithic piezoelectric control of soliton microcombs. http://arxiv.org/abs/1912.08686 (2019). 10.1038/s41586-020-2465-8 32669694

[48] Dong, B., Tian, H., Zervas, M., Kippenberg, T. J. & Bhave, S. A. Port: A piezoelectric optical resonance tuner. In *2018 IEEE Micro Electro Mechanical Systems (MEMS)* c, 739–742 (IEEE, 2018). 10.1109/MEMSYS.2018.8346661.

[49] Alexander K (2018). Nanophotonic pockels modulators on a silicon nitride platform. Nat. Commun..

[50] He Y (2019). Self-starting bi-chromatic linbo 3 soliton microcomb. Optica.

[51] Kéfélian F, Jiang H, Lemonde P, Santarelli G (2009). Ultralow-frequency-noise stabilization of a laser by locking to an optical fiber-delay line. Opt. Lett..

[52] Liang W (2015). Ultralow noise miniature external cavity semiconductor laser. Nat. Commun..

[53] Gundavarapu S (2019). Sub-hertz fundamental linewidth photonic integrated brillouin laser. Nat. Photonics.

[54] Marpaung D, Yao J, Capmany J (2019). Integrated microwave photonics. Nat. Photonics.

[55] Ghelfi P (2014). A fully photonics-based coherent radar system. Nature.

[56] Koenig S (2013). Wireless sub-thz communication system with high data rate. Nat. Photonics.

[57] Lucas E, Guo H, Jost JD, Karpov M, Kippenberg TJ (2017). Detuning-dependent properties and dispersion-induced instabilities of temporal dissipative kerr solitons in optical microresonators. Phys. Rev. A.

[58] Yi X, Yang Q-F, Yang KY, Vahala K (2016). Theory and measurement of the soliton self-frequency shift and efficiency in optical microcavities. Opt. Lett..

[59] Yi X (2017). Single-mode dispersive waves and soliton microcomb dynamics. Nat. Commun..

[60] Matsko AB, Maleki L (2013). On timing jitter of mode locked kerr frequency combs. Opt. Express.

[61] Herr T (2012). Universal formation dynamics and noise of kerr-frequency combs in microresonators. Nat. Photonics.

[62] Yang Q-F, Yi X, Yang KY, Vahala K (2016). Spatial-mode-interaction-induced dispersive waves and their active tuning in microresonators. Optica.

[63] Bao C (2017). Soliton repetition rate in a silicon-nitride microresonator. Opt. Lett..

[64] Wilson Dalziel J., Schneider Katharina, Hönl Simon, Anderson Miles, Baumgartner Yannick, Czornomaz Lukas, Kippenberg Tobias J., Seidler Paul (2019). Integrated gallium phosphide nonlinear photonics. Nature Photonics.

[65] Gorodetsky ML, Grudinin IS (2004). Fundamental thermal fluctuations in microspheres. J. Optical Soc. Am. B.

[66] Guo H (2017). Universal dynamics and deterministic switching of dissipative kerr solitons in optical microresonators. Nat. Phys..

[67] Zhang W (2011). Amplitude to phase conversion of ingaas pin photo-diodes for femtosecond lasers microwave signal generation. Appl. Phys. B.

[68] Niebauer TM, Schilling R, Danzmann K, Rüdiger A, Winkler W (1991). Nonstationary shot noise and its effect on the sensitivity of interferometers. Phys. Rev. A.

[69] Quinlan F, Fortier TM, Jiang H, Diddams SA (2013). Analysis of shot noise in the detection of ultrashort optical pulse trains. J. Opt. Soc. Am. B.

[70] Quinlan F (2013). Exploiting shot noise correlations in the photodetection of ultrashort optical pulse trains. Nat. Photonics.

[71] Giunta, M. et al. Transportable ultra-low noise photonic microwave synthesizer. In *Conference on Lasers and Electro-Optics*, OSA Technical Digest (online) p. SM2L.5 (Optical Society of America, San Jose, California, 2018) 10.1364/CLEO_SI.2018.SM2L.5.

