# Supplementary material for: Ultralow-noise photonic microwave synthesis using a soliton microcomb-based transfer oscillator
Source: Nat Commun. 2020 Jan 17;11:374. doi: 10.1038/s41467-019-14059-4 (PMC6969110; doi:10.1038/s41467-019-14059-4)
Supplement: Supplementary file 1 — Supplementary Information [file 41467_2019_14059_MOESM1_ESM.pdf]

Supplementary Information for: Ultralow-Noise Photonic Microwave Synthesis using a Soliton Microcomb-based Transfer Oscillator

Erwan Lucas,[1,*] Pierre Brochard,[2,*] Romain Bouchand,[1]
Stéphane Schilt,[2] Thomas Südmeyer,[2] and Tobias J. Kippenberg[1]

[1]*Institute of Physics, École Polytechnique Fédérale de Lausanne (EPFL), CH-1015 Lausanne, Switzerland*
[2]*Laboratoire Temps-Fréquence, Université de Neuchâtel, CH-2000 Neuchâtel, Switzerland*

Supplementary Note 1: Soliton generation procedure and optical phase lock loop

In a microresonator with appropriate dispersion, solitons emerge spontaneously (soft excitation) when the continuous wave pump laser is scanned from blue (higher laser frequency) to red detuning (lower laser frequency) across a high-Q resonance[1]. This requires a rapid tunability of either the pump laser or the resonator. An ultra-stable laser (USL) locked to a high-finesse reference cavity is typically not tunable and the crystalline resonator used here cannot be tuned fast enough to overcome thermal effects[2]. We circumvented this problem by implementing an optical phase lock loop (OPLL) to stabilise the frequency offset between the pump external-cavity diode laser (ECDL, Toptica CTL1550) and the reference USL (Menlo Systems ORS1500). The soliton state is generated by rapid tuning of the ECDL via current tuning. After soliton generation, the OPLL is activated with a frequency offset set so as to preserve the soliton. Resonators with improved tuning capabilities could remove the need for an OPLL altogether.

Solitons are sustained in the cavity within a narrow range of 'red' laser–cavity detuning[1,3] (i.e., the laser frequency is lower than the resonance frequency), which depends on the pump power level and the coupling rate of the resonator. The detuning control is crucial in soliton-based Kerr comb as it determines many of the comb properties. First, in order to preserve the soliton in the cavity, the detuning must remain within the soliton existence range. Secondly, as exposed in the Methods section, the soliton properties, such as the pulse duration and especially the repetition rate, are highly sensitive to the detuning operating point[4,5]. However, even if an USL is used to pump the resonator, the thermal drift of the resonator induces detuning drifts that can lead to degraded noise performance and even loss of the soliton. Therefore, following soliton generation and OPLL activation, an offset Pound-Drever-Hall (PDH) lock actively stabilises the detuning to a precise, defined radio-frequency (RF).

The schematic of the OPLL is presented in Supplementary Fig. 1a. The beatnote between the two lasers is photo-detected at a frequency of 1.7 GHz, and down-mixed to an intermediate frequency (IF) of 20 MHz using a frequency synthesiser. After soliton generation, the synthesiser frequency is set precisely using a frequency counter, in order to preserve the pump laser within the soliton existence range upon lock activation. The IF signal is band-pass filtered and compared to a 20 MHz RF signal derived from a 10 MHz common clock, using a double-balanced mixer (DBM). The resulting error signal passes through a proportional–integral–derivative controller (PID, Toptica MFALC) that implements a slow feedback to the laser piezoelectric transducer and a fast feedback to the diode laser current allowing a ∼ 500 kHz actuation bandwidth (see Supplementary Fig. 1b). The residual noise of the OPLL and the phase noise of the

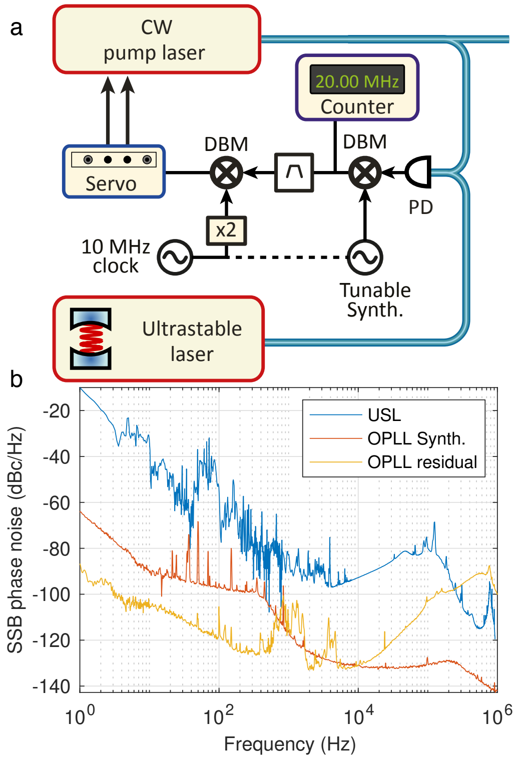

Supplementary Figure 1: **Optical phase lock loop** **(a)** Detailed experimental setup. PD, photodiode; DBM double balanced mixer; ×2, frequency doubler. **(b)** Comparison between the USL phase noise and the residual phase noise contribution of the OPLL components. The red line shows the phase noise of the 1.7 GHz synthesiser and the yellow line the phase noise of the 20 MHz IF signal at the output of the DBM, indicating a feedback bandwidth in the range of ∼ 500 kHz. The overall added noise is negligible compared to the USL.

* These authors contributed equally to this work.

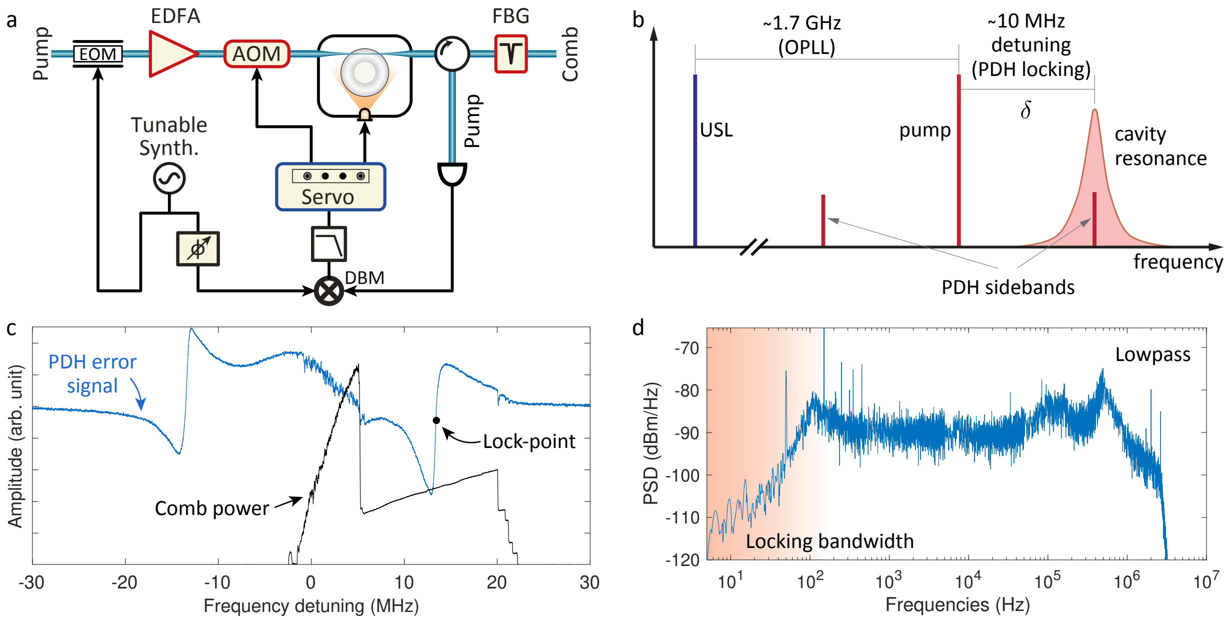

Supplementary Figure 2: **Pound-Drever-Hall detuning stabilisation (a)** Detailed experimental setup. EOM, electro-optic modulator; EDFA, erbium-doped fibre amplifier; AOM, acousto-optic modulator; FBG, fibre Bragg grating. **(b)** Scheme of principle of the stabilisation. The pump laser is phase-locked to the USL. The cavity resonance detuning is then locked to the pump laser via PDH stabilisation using a feedback to the pump laser power (and radiative heating of the resonator). **(c)** PDH error signal (blue) and generated comb power (black), as a function of detuning (the PDH frequency is 13.4 MHz). The detuning lock-point can be set arbitrary in the soliton step by changing the synthesiser frequency. **(d)** Power spectral density (PSD) of the residual PDH error signal, when the detuning lock is active.

synthesiser are negligible compared to the USL noise, indicating a good transfer of the USL purity to the pump laser (see Supplementary Fig. 1b).

Supplementary Note 2: Resonator stabilisation

The detuning stabilisation is implemented via a PDH stabilisation (see Supplementary Fig. 2). The PDH error signal is obtained by phase-modulating the pump laser (at a frequency in the range of 5 – 25 MHz) before coupling to the cavity, using an electro-optic modulator (EOM, iXblue MPX-LN-0.1). The modulated signal is detected after the resonator (on the filtered residual pump) and demodulated to DC using the same phase-shifted RF signal (to account for the unbalanced delay between the modulation and demodulation paths). In practice, a dual-channel arbitrary waveform generator is used and the relative phase between the channels is adapted as a function of the modulation frequency. After demodulation, the baseband signal is low-pass-filtered and sent to a PID servo-controller (Toptica FALC). When the comb operates in the soliton regime, the pump laser is red-detuned. Thus, the PDH feedback setpoint corresponds to the higher frequency phase modulation sideband being in resonance (Supplementary Fig. 2b). The PDH servo acts thermally on the resonator, to maintain a fixed pump-resonator detuning. The feedback is implemented to the pump laser power (using a 0th order acousto-optic modulator (AOM)) and a slower actuation on a LED (Thorlabs MCWHL5 with a typical power of 800 mW) shining on the resonator through a microscope also used for imaging. This allows keeping the pump power to a determined setpoint. The residual noise of the PDH error signal indicates an actuation bandwidth of ~ 100 Hz, limited by the thermal response of the resonator (Supplementary Fig. 2d).

Owing to this overall scheme, the resonator is stabilised to the USL, which improves the stability of the system and helps preserving a given operation point.

Supplementary Note 3: Auxiliary comb and offset detection

An auxiliary optical frequency comb (Menlo Systems FC1500) is used here to detect the CEO frequency of the Kerr-comb $f_{\text{CEO}}^{\text{K}}$, as the used crystalline micro-comb features a relatively narrow spectrum that prevents a direct detection of its CEO frequency. An optical beat-note between the two combs is first detected with a photodiode (NewFocus model 1811) at a frequency of a few tens of MHz. This low-frequency beat signal corresponds to the frequency difference between one mode of each comb, i.e.,

$$f_{\text{beat}} = N(f_{\text{rep}}^{\text{K}} - 56\, f_{\text{rep}}^{\text{aux}}) + (f_{\text{CEO}}^{\text{K}} - f_{\text{CEO}}^{\text{aux}}) \qquad (1)$$

where the superscripts 'K' and 'aux' refer to the Kerr and auxiliary comb, respectively, and the 56th harmonic

of the 251.6 MHz repetition rate of the auxiliary comb is in close vicinity to the fundamental repetition rate of the Kerr comb.

Supplementary Note 4: Injection locking

To suppress the relative phase noise between the repetition rate of the two combs in their beat signal, we imprint the $f_{\rm rep}$ noise of the auxiliary comb to the Kerr comb by injection locking. This is realised by detecting and band-pass filtering the 56$^{\rm th}$ harmonic of $f_{\rm rep}^{\rm aux}$ (auxiliary comb) at 14.09 GHz and using this signal, after amplification to $\sim$ 19 dBm, to drive an EOM (iXblue MPZ-LN-10) to create a set of sidebands around the ultra-stable pump laser of the micro-resonator, which injection-lock the adjacent optical modes of the resonator. This strongly correlates the noise of the repetition rate of the two combs, but only within the bandwidth of the injection locking that is in the kHz range, as shown in Supplementary Fig. 3. In that case, the beat signal frequency in eq. (1) can be re-expressed as

$$f_{\rm beat} = f_{\rm CEO}^{\rm K} - f_{\rm CEO}^{\rm aux} = \Delta f_{\rm CEO} \qquad (2)$$

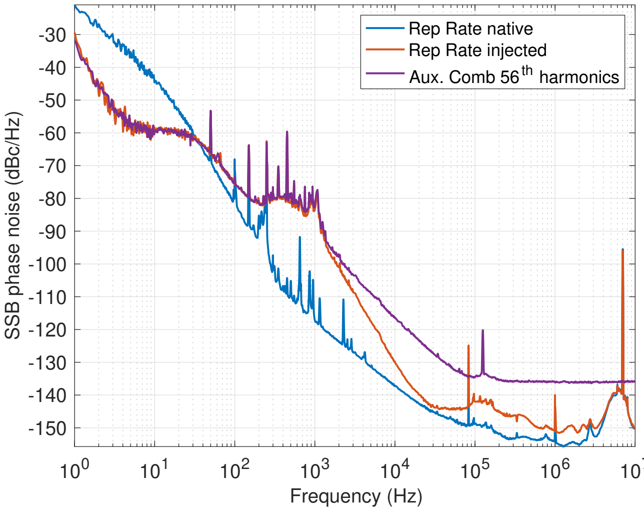

Supplementary Figure 3: **Injection locking of the repetition rate** Comparison between the phase noise of the Kerr comb repetition rate when it is native (blue) and injection locked (red) by the 56$^{\rm th}$ harmonic of the auxiliary comb repetition rate (purple).

Supplementary Note 5: Division chain

The transfer oscillator approach is implemented in this work with a 2-DDS scheme as introduced by Brochard and co-authors[6] to perform electronic division with a finely adjustable ratio. This implementation also makes possible the generation of a low-noise single-tone RF output signal by efficiently filtering out other spurious peaks that would occur with a single DDS. The practical realisation of this scheme with the Kerr comb is depicted in Supplementary Fig. 4.

The beat signal $\Delta f_{\rm CEO}$ between the two combs is mixed in a double balanced mixer (DBM) with the CEO signal of the auxiliary comb $f_{\rm CEO}^{\rm aux}$ (detected using a standard $f - 2f$ interferometer) in order to remove this contribution and retrieve $f_{\rm CEO}^{\rm K}$. Prior to this mixing, the CEO signal of the auxiliary comb, which is stabilised at 20 MHz, is frequency-up-shifted using a low-noise synthesiser. This provides a straightforward means to arbitrarily tune $f_{\rm CEO}^{\rm aux}$ without changing it optically (which would also change the optical beat-note frequency by $\Delta f_{\rm CEO}$) and without frequency noise degradation, as the measured phase noise of the intermediate synthesiser is comparatively negligible. Thereby, the effective sign of $f_{\rm CEO}^{\rm aux}$ can be quickly changed, without changing any RF component, in order to properly remove its contribution when mixing with $\Delta f_{\rm CEO}$ in the DBM.

Furthermore, this additional flexibility enables us to finely adjust the signal frequency at the DBM output, which contains the effective Kerr comb CEO, to make it coincide with the frequency of a tracking oscillator around 40 MHz. This tracking oscillator consists in a narrow-band low-noise voltage-controlled oscillator (VCO) that is phase-locked to $f_{\rm CEO}^{\rm K}$. The fine adjustment of the VCO locking bandwidth (by tuning the PI parameters of the feedback) enables us to filter the noise of the detected $f_{\rm CEO}^{\rm K}$ in order to match the $\sim$ 2 kHz injection locking bandwidth over which the relation $f_{\rm rep}^{\rm K} = 56 f_{\rm rep}^{\rm aux}$ is ensured. Thereby, the contribution of the residual uncorrelated fluctuations between the two combs in the generated ultralow-noise RF signal is minimised. Lower ($\sim$ 200 Hz) or higher ($\sim$ 20 kHz) feedback bandwidths lead to an increased noise in the final signal at low or high Fourier frequencies respectively (Supplementary Fig. 5) as a result of the imperfect compensation of the auxiliary comb noise. Furthermore, as an RF signal with a sufficient signal-to-noise ratio (SNR) of more than 30 dB is needed for a proper and stable operation of the subsequent frequency divider, this tracking oscillator helps improving the signal quality, so that even a fairly low SNR of the signal at the output of the DBM makes possible to implement the transfer oscillator method.

The signal after the tracking oscillator is up-converted to 15 GHz using a synthesiser with an absolute phase noise lower than the USL (see Supplementary Fig. 6), so that its noise has a negligible contribution in the final signal. This frequency shift is necessary to perform the subsequent frequency division by a large number of around 13,698. Eventually, this synthesiser could be replaced by the repetition rate of the Kerr-comb to alleviate any of the associated limitation. This was not implemented here due to the lack of appropriate filters.

The frequency division from 15 GHz to 1.095 MHz is realised first by a frequency pre-scaler ($\div$6, RF Bay FPS-6-15) followed by two DDS (AD9915 evaluation board) in parallel that respectively output signals at 100 MHz and 101.095 MHz from their 2.5 GHz input clock signal. These two signals are subsequently mixed with the repetition rate of the Kerr comb separately detected using a

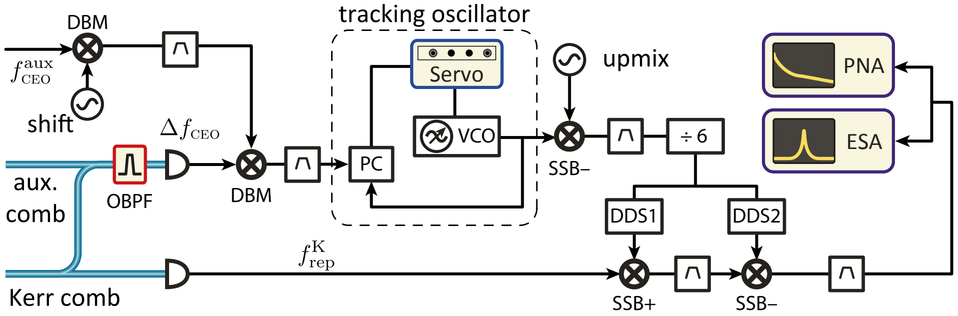

Supplementary Figure 4: **Frequency chain for the transfer oscillator division** Implementation of the optical-to-microwave frequency division using the 2-DDS transfer oscillator scheme. The f_{rep} component of the Kerr comb detected with a fast photodiode (lower path) is mixed with the CEO signal frequency-divided by a factor N (upper path). The division from 15 GHz to 1.095 MHz is realised by a frequency pre-scaler (÷6) followed by two DDS in parallel that output signals at 100 MHz and 101.095 MHz, respectively, from the 2.5 GHz input clock signal. The CEO frequency of the Kerr comb is indirectly obtained from the subtraction of the frequency-shifted auxiliary self-referenced comb CEO $f_{\mathrm{CEO}}^{\mathrm{aux}}$ with the optical beat-note from the two combs (Δf_{CEO}), due to the fact that the phase noise of the repetition rate of the two combs is correlated by an injection locking scheme. DBM, double-balanced mixer; VCO, voltage-controlled oscillator; PC, digital phase comparator; SSB(+/-), single sideband mixer (sum/difference frequency); DDS, direct digital synthesiser; PNA, phase noise analyser; ESA, electrical spectrum analyser.

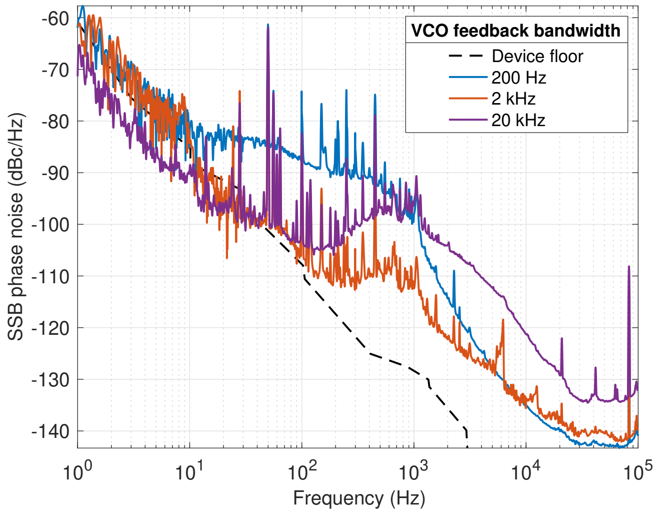

Supplementary Figure 5: **Tracking oscillator effect.** Influence of the feedback bandwidth of the tracking oscillator, used to filter the Kerr comb f_{CEO}, onto the final generated RF signal. The lowest phase noise of the final RF signal is obtained for a locking bandwidth of the VCO of ~ 2 kHz (red curve), similar to the bandwidth of the injection locking process used to correlate the noise of the repetition rate of the two combs. A lower (~ 200 Hz, blue curve) or higher (~ 20 kHz, purple curve) bandwidth results in a higher noise of the generated RF signal.

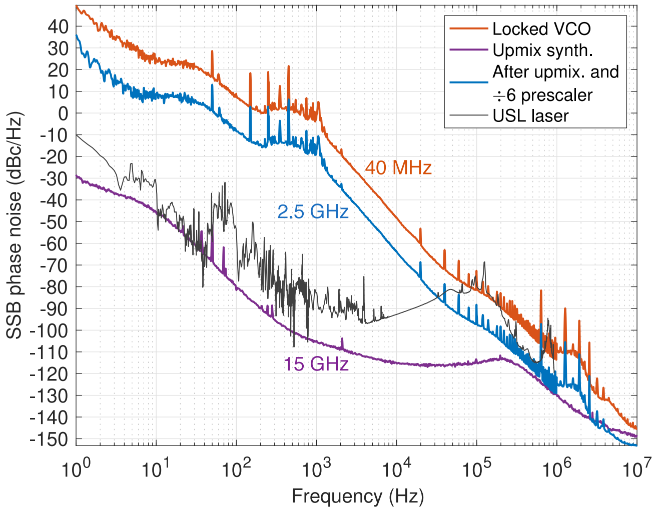

Supplementary Figure 6: **Upmixing and pre-scaler division.** Evolution of the signal phase noise during the upmixing and pre-scaler division of the filtered Kerr comb CEO. The carrier frequencies are indicated on the plot. The noise of the upmixing synthesiser (purple curve) is lower than the USL noise (black), such that it does not limit the final division result. Upmixing with the comb repetition rate would avoid this potential limitation.

fast photodiode (Discovery Semiconductors DSC40) and filtered to select the proper component that corresponds to $f_{\mathrm{RF}} = f_{\mathrm{rep}}^{\mathrm{K}} + f_{\mathrm{CEO}}^{\mathrm{K}}/N = \nu_{\mathrm{USL}}/N$. The sequential mixing followed by filtering after each DDS allows for the efficient rejection of the spurious peaks occurring at harmonics of the DDS signals, thanks to their relatively high frequency spacing (100 MHz range).

The generated ultralow noise RF signal is characterised using a phase noise analyser (PNA, model FSWP26 from Rohde-Schwarz) and an electrical spectrum analyser (ESA, model FSW43 from Rohde-Schwarz). The effect of

the frequency division performed with the 2-DDS scheme is illustrated in Supplementary Fig. 7. The frequency difference of the two signals at 100 MHz and 101.095 MHz, respectively, gives a signal at 1.095 MHz which correspond to $f_{\mathrm{CEO}}^{\mathrm{K}}/N$ and is strongly correlated with $f_{\mathrm{rep}}^{\mathrm{K}}$ at 14.09 GHz. Mixing these two signal results in the generation of a very low noise RF signal that demonstrates a high rejection of the Kerr comb phase noise.

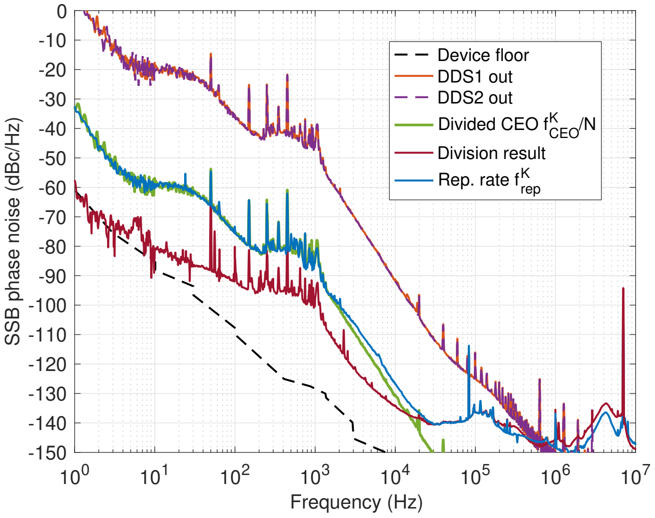

Supplementary Figure 7: **Noise division demonstration** Demonstration of the noise division achieved by the 2-DDS scheme. The orange and dashed violet curves show the phase noise PSD separately measured at the output of each DDS at 101.095 MHz (DDS1) and 100 MHz (DDS2), respectively. The green curve displays the noise of the frequency-divided CEO signal of the Kerr comb, which overlaps the noise of the repetition rate (blue curve). Therefore, these two noise contributions compensate each other to a large extend in the final RF signal (red curve), which demonstrates the noise improvement brought by the transfer oscillator scheme limited here by the Kerr comb injection locking bandwidth.

Supplementary Note 6: Sign effect in the transfer oscillator

The high rejection of the Kerr comb phase noise offered by the transfer oscillator scheme requires mixing signals with the proper sign combination, so that the frequency fluctuations of $f_{\mathrm{rep}}^{\mathrm{K}}$ and $f_{\mathrm{CEO}}^{\mathrm{K}}$ are indeed compensated in the final RF signal. The sign of $f_{\mathrm{CEO}}^{\mathrm{K}}$ is determined by the heterodyne beat between the Kerr comb and the auxiliary comb, which can be controlled by the repetition rate of the auxiliary comb, and by the subtracted CEO signal of the auxiliary comb, whose sign can be changed using the frequency-shifting synthesiser as previously mentioned. The sign of the $f_{\mathrm{rep}}^{\mathrm{K}}$ contribution to be removed can be adjusted by inverting the output frequencies of the two DDS, without changing any RF component. This is illustrated in Supplementary Fig. 8, which shows how the noise is correctly compensated with the proper sign and increases by a factor of 4 (in terms of PSD, or +6 dB) compared to the noise of f_{rep} with the incorrect sign (as the resulting signal corresponds to $\nu_{\mathrm{USL}}/N + 2f_{\mathrm{rep}}$).

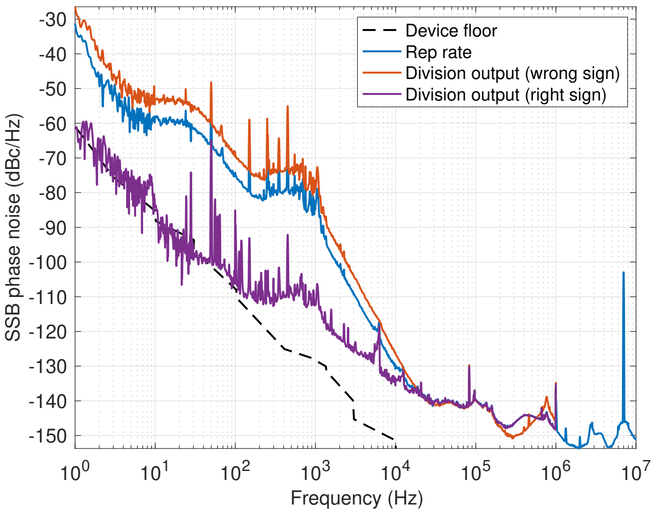

Supplementary Figure 8: **Sign effect** Demonstration of the adjustment of the sign of the correction of the Kerr comb $f_{\mathrm{rep}}^{\mathrm{K}}$ noise in the low-noise RF output signal generated by the transfer oscillator scheme. The blue curve displays the phase noise of the Kerr comb repetition rate. The purple curve shows the phase noise of the generated RF signal obtained with the correct sign where the $f_{\mathrm{rep}}^{\mathrm{K}}$ noise is removed, whereas the orange curve corresponds to the other sign (obtained by inverting the frequency of the two DDS), which leads to a 6 dB noise increase (the output signal contains twice the frequency fluctuations of $f_{\mathrm{rep}}^{\mathrm{K}}$).

Supplementary Note 7: Source of imperfect noise compensation

The noise compensation in the transfer oscillator method relies on the subtraction of various noise contributions of the micro-resonator comb. Perfect noise compensation occurs when no relative delay is introduced between f_{CEO}/N and f_{rep} at the time of their final mixing. If a significant delay occurs between the signals, the noise compensation may be incomplete as the signals become imperfectly correlated[7] and the residual (uncompensated) noise scales according to

$$S_{\varphi}^{\mathrm{signal}}(f) = \frac{1}{N^2}\, S_{\varphi}^{\mathrm{USL}}(f) + 4\sin^2(\pi\tau f)\; S_{\varphi}^{\mathrm{rep}}(f)\,, \qquad (3)$$

where τ is the relative delay, f is the Fourier frequency and S_{φ} denotes the phase noise power spectral density. If $\tau = 0$ the phase noise of the repetition rate is properly cancelled. Some care is thus needed to minimise the delays, in order to maximise the noise cancellation bandwidth, but this factor is not critical. For example,

a coarse length mismatch of 10 m would correspond to a delay of $\sim$ 42 ns (assuming a velocity factor of 80 %), resulting in a fully uncompensated noise (0 dB rejection) reached at a Fourier frequency of $\sim$ 4 MHz.

Supplementary References

[1] T. Herr, V. Brasch, J. D. Jost, C. Y. Wang, N. M. Kondratiev, M. L. Gorodetsky, and T. J. Kippenberg, "Temporal solitons in optical microresonators," Nature Photonics **8**, 145–152 (2013).

[2] Tal Carmon, Lan Yang, and Kerry J. Vahala, "Dynamical thermal behavior and thermal self-stability of microcavities," Optics Express **12**, 4742 (2004).

[3] F Leo, S Coen, P Kockaert, S-P Gorza, P Emplit, and M Haelterman, "Temporal cavity solitons in one-dimensional kerr media as bits in an all-optical buffer," Nature Photonics **4**, 471–476 (2010).

[4] Xu Yi, Qi-Fan Yang, Xueyue Zhang, Ki Youl Yang, Xinbai Li, and Kerry Vahala, "Single-mode dispersive waves and soliton microcomb dynamics," Nature Communications **8**, 14869 (2017).

[5] Jordan R. Stone, Travis C. Briles, Tara E. Drake, Daryl T. Spencer, David R. Carlson, Scott A. Diddams, and Scott B. Papp, "Thermal and nonlinear dissipative-soliton dynamics in kerr-microresonator frequency combs," Physical Review Letters **121**, 063902 (2018).

[6] Pierre Brochard, Stéphane Schilt, and Thomas Südmeyer, "Ultra-low noise microwave generation with a free-running optical frequency comb transfer oscillator," Optics Letters **43**, 4651–4654 (2018).

[7] Enrico Rubiola, Ertan Salik, Shouhua Huang, Nan Yu, and Lute Maleki, "Photonic-delay technique for phase-noise measurement of microwave oscillators," Journal of the Optical Society of America B **22**, 987 (2005).
